# Outer Membrane Vesicles from Caulobacter crescentus: A Platform for Recombinant Antigen Presentation

**DOI:** 10.1021/acsnano.4c17885

**Published:** 2025-05-28

**Authors:** Luis David Ginez, Aurora Osorio, Víctor Correal-Medina, Thelma Arenas, Claudia González-Espinosa, Laura Camarena, Sebastian Poggio

**Affiliations:** † Departamento de Biología Molecular y Biotecnologia, Instituto de Investigaciones Biomédicas, Universidad Nacional Autónoma de México, Instituto de Investigaciones Biomédicas, Circuito Mario de la Cueva s/n, Ciudad Universitaria, Ciudad de México 04510, México; ‡ Pharmacobiology Department and Center for Research in Aging, Center for Research and Advanced Studies (Cinvestav), South Campus, Calzada de los Tenorios No. 235, Col. Granjas Coapa, Alcaldía Tlalpan, Ciudad de México 14330, México

**Keywords:** outer membrane vesicles, Caulobacter crescentus, S-layer, OmpA, vaccine platform, lipid A, inflammatory cytokines

## Abstract

Bacterial outer membrane vesicles (OMVs) are an emerging and attractive technology for the generation of vaccines. Their properties as natural adjuvants, size, acellularity, and comparative cost of production favor their use as vaccines. Two major caveats for the use of OMVs as vaccines are their biological safety, since OMVs can induce a severe and even fatal inflammatory response and that they are naturally produced in low amounts. In this study, we show that a strategy to induce the production of OMVs applied to the nonpathogenic bacterium Caulobacter crescentus results in a strain with good OMV yields. In comparison with the OMVs derived from Escherichia coli K-12, the OMVs from C. crescentus induce a lower inflammatory response in an *in vivo* murine model of acute inflammation and in a human cell assay. Also, only minor signs of pain in mice were observed even at high doses. The C. crescentus OMVs can be efficiently loaded with a recombinant protein and induce antibody production against it with an adjuvant effect, indicating that these OMVs are viable vehicles for the presentation of recombinant antigens. These results support the use of the OMVs obtained from C. crescentus as a safe and effective platform for the development of low-cost vaccines.

## Graphical abstract

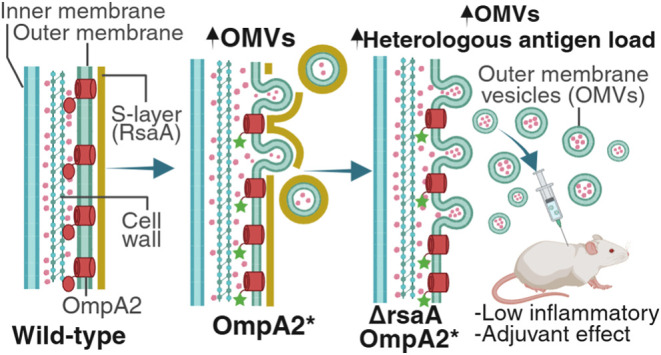

Bacterial outer membrane vesicles (OMVs) are small nanoparticles (25–200 nm) produced during growth by Gram-negative bacteria either naturally or by treatment or mutation. OMVs harbor numerous membrane-associated proteins as lipoproteins or integral outer membrane proteins (OMPs), while in their lumen, they carry soluble periplasmic proteins and other compounds including fragments of the cell wall and occasionally cytoplasmic and inner membrane proteins or nucleic acids.^1,2^ OMVs are produced under diverse stimuli and stress conditions, such as nutrient deprivation, exposition to antibiotics, bacteriophages, and changes in temperature or pH.^3,4^ For pathogenic bacteria, OMVs can help evade or modulate the immune response or function as active elements of the pathogenic process.^5–9^ Since OMVs carry many of the antigens present on the bacterial surface without being replicative, OMVs have been explored as potential vaccines against the bacteria from which they are obtained and have been shown to induce protective immunity.^3^ The presence of a diverse array of antigens in their native conformation endows OMVs with natural immunogenicity.^2^ In addition, some of their components including lipopolysaccharides (LPS), lipoproteins, OMPs, peptidoglycan fragments, and flagellins have motifs known as pathogen-associated molecular patterns (PAMPs) that act as adjuvants by inducing the production of inflammatory cytokines by the innate immune system.^10,11^ The OMV size allows them to disperse through the lymphatic system, and their efficient uptake and processing by antigen-presenting cells (APCs) further support their use for vaccine development.^12^ Yet, only a few OMV-based vaccines are currently licensed, primarily targeting Neisseria meningitidis serogroup B and Haemophilus influenzae type b, with other candidates in clinical development.^13^ One main concern about using OMVs as vaccines is their biological safety given their propensity to trigger an excessive activation of the innate immune response, resulting in life-threatening systemic inflammation.^8,14^ Genetic and chemical strategies have been developed to reduce OMV toxicity, mainly based on the modification of the LPS.^15–17^ Alternatively, detergent treatment reduces the LPS content from the vesicles but can also remove relevant antigens.^18^ Despite using these strategies, approved vaccine formulations have to include substances that regulate the release of OMVs to modulate the initial inflammatory response.^3^ Another limiting factor for the use of OMVs as vaccines is that bacteria naturally produce a small amount of these nanoparticles, complicating their purification. To increase yields, a variety of physical and chemical treatments as well as genetic modifications are currently employed. Several mutations, mainly based on genes present in Escherichia coli and other γ proteobacteria, have been reported to increase the production of OMVs. The genes affected by these mutations are involved in different conserved mechanisms related to the maintenance of the cell wall or outer membrane.^18,19^ One of such strategies is to weaken the interaction between the OM and the cell wall as it occurs in strains lacking Braun’s lipoprotein (Lpp), the integral outer membrane protein OmpA, or the proteins of the Tol-Pal system.^20–22^ A different strategy involves reducing protein quality control in the periplasm by eliminating the dual-function DegP chaperone/protease. This alteration leads to the accumulation of misfolded proteins in this compartment, consequently promoting the overproduction of OMVs, especially at high temperatures.^4^ Another blank to induce the formation of OMVs in E. coli is the *nlpI* gene, the product of which is involved in cell division and regulates peptidoglycan synthesis and degradation.^23^ In bacteria that do not have homologous proteins with those known to be involved in the stability of the cell envelope, target genes need to be identified. This was the case for N. meningitidis, in which the OMV production is increased by eliminating the peptidoglycan-binding outer membrane protein RmpM.^24^ An additional risk of OMVs obtained from pathogenic bacteria comes from the presence of pathogenically active proteins that can reproduce some of the symptoms of an active infection.^8^ Instead of OMVs from the pathogen of interest, relevant antigens can be incorporated into the OMVs produced by a different bacterium that could serve as a safe platform for their expression. This strategy has not only been used with pathogenic bacteria for which OMV-producing strains have been developed but also different nonpathogenic E. coli strains have been reported with this purpose.^25,26^

Caulobacter crescentus is a nonpathogenic Gram-negative oligotrophic bacterium.^27^ Wild-type C. crescentus cells are covered by an S-layer, a protein layer that in this bacterium consists of the RsaA protein that binds to the O-antigen of the LPS forming a semicrystalline array. At one of their poles, C. crescentus cells grow a stalk, a thin tubular extension of the three layers of the cell envelope that in some conditions can reach several times the cell length. The LPS from this bacterium has several relevant structural differences from that of enteric bacteria,^28^ which reduce its endotoxicity. These differences include the absence of phosphate groups in the lipid A moiety and unsaturation in some of its six fatty acyl chains. Consequently, its inflammatory activity on murine macrophages is about 100-fold lower than that of truncated E. coli LPS.^29^ The use of C. crescentus for therapeutic and prophylactic treatments against some diseases^30,31^ and the design of recombinant vaccines using purified recombinant proteins or even whole, live, or heat-killed bacteria^32–37^ has been proposed. Since even whole C. crescentus cells show low reactogenicity, OMV from this bacterium could be a good alternative. We previously reported that C. crescentus has two OmpA homologues of which only OmpA2 is a highly abundant protein that is required for OM stability.^38^ A strain that expresses a fluorescent version of OmpA2 harboring a single amino acid substitution (R351A) in its C-terminal domain produces abundant OMVs.^39^ Based on these previous results, in this work, we generated a strain with a high yield of OMVs. These vesicles have a higher concentration of soluble proteins and an improved relative concentration of a recombinant protein. We also developed a system that allows the constitutive expression of a recombinant antigen and its targeting to the vesicular lumen. Immunological characterization of these vesicles showed that they induce low levels of inflammatory cytokines in both mice and human models of acute inflammation. Notably, these OMVs induce antibody production against a recombinant protein contained in their lumen more efficiently than does the recombinant protein alone.

## Results

### The S-Layer Protein RsaA is a Major Component of the OMVs from C. crescentus

In a previous work, we noticed that a C. crescentus strain expressing the OmpA2_R351A_–mCherry protein fusion produced OMVs.^39^ To determine if this effect was due to the combination of the amino acid substitution and the fusion with mCherry, we decided to obtain a strain with only the OmpA2_R351A_ coding allele. Replacement of the wild-type *ompA*2 gene by the mutant allele resulted in strain CVOM-1 (Caulobacter Vesicles Outer Membrane), and staining CVOM-1 cells with the membrane dye FM4–64FX showed the presence of OMVs (Figure S1 of the Supporting Information). an integrative plasmid that constitutively expresses a periplasmic version of the mCherry fluorescent protein from a strong native promoter (Figure 1A). For this, the promoter of the *rsaA* gene was selected since the *rsaA* transcript and protein are highly abundant and the *rsaA* promoter is a strong promoter that is active throughout the cell cycle.^40^ To mediate the translocation to the periplasm of the mCherry protein, we added the signal peptide of the C. crescentus DipM protein and a short linker region from this protein^41^ in-frame with the N-terminus of mCherry. When this construct was integrated into the chromosome of the wild-type CB15N strain, strong periplasmic fluorescence could be observed (Figure 1A). Observation of a culture sample of wild-type and CVOM-1 cells expressing the periplasmic mCherry confirmed the presence of OMVs in the CVOM-1 cultures (Figure 1A). The OMVs from CVOM-1 cells not expressing the periplasmic mCherry were purified, and their protein profile was examined in a denaturing SDS-PAGE. This showed that the most abundant protein in these particles has an apparent molecular weight of 100 kDa, which we presumed corresponded to the S-layer protein RsaA (Figure 1B). To probe this idea, we generated the CVOM-2 strain by eliminating the coding sequence for *rsaA* from the chromosome of the CVOM-1 strain. The absence of RsaA did not affect cell morphology, but unexpectedly, we observed an apparent increase in OMV production in the CVOM-2 strain (Figures 1A and S1 of the Supporting Information). This effect was not detected in a strain carrying only the Δ*rsaA* mutation (Figure 1A). Much brighter OMVs were also observed in the CVOM-2 samples expressing periplasmic mCherry, which together with a lower fluorescence from the cells suggests that the OMVs from this strain are more efficiently loaded with the periplasmic mCherry.

**Figure 1:**
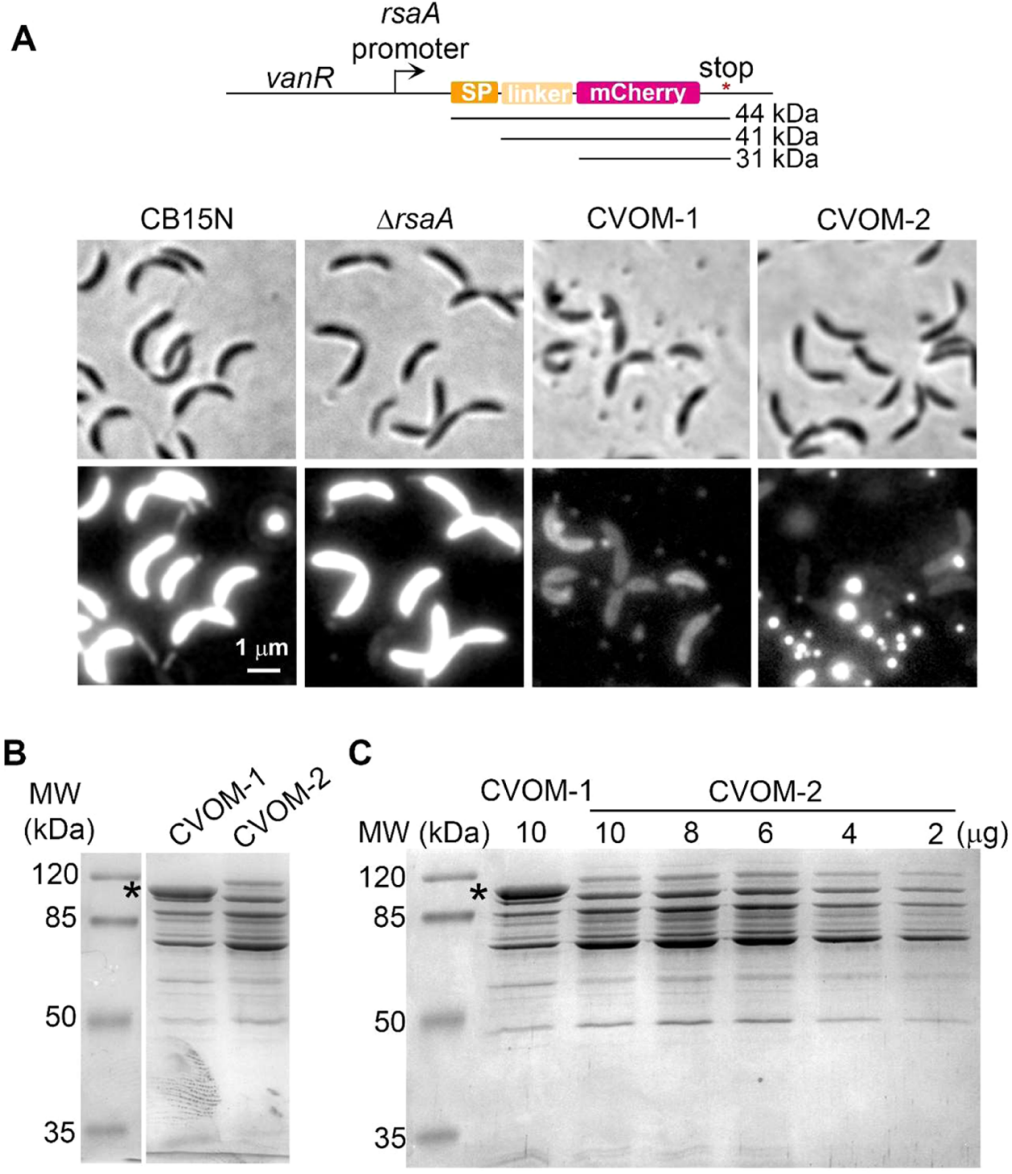
RsaA is the major protein in the C. crescentus OMVs, and its absence increases OMV production. (A) Map of the construct for constitutive expression of mCherry from the C. crescentus chromosome. SP: signal peptide of the DipM protein to mediate the translocation of mCherry to the periplasm; linker: first 87 amino acids of the DipM protein after the predicted maturation site. The predicted molecular weights of the three possible resulting polypeptides are indicated. Below, micrographs show the cell morphology and presence of the OMVs in different strains constitutively expressing periplasmic mCherry. Strains CB15N, Δ*rsaA* (SP1587), CVOM-1 (SP1569), and CVOM-2 (SP1570). Brightness and contrast are the same for all images. (B) SDS-PAGE of purified OMVs from the indicated strains; the asterisk indicates the band corresponding to the RsaA protein (∼100 kDa). (C) SDS-PAGE of purified OMVs from the CVOM-1 and CVOM-2 strains. To compare the protein profiles, the indicated decreasing quantities of the CVOM-2 vesicles were loaded. The asterisk indicates the band corresponding to the RsaA protein.

As expected, the protein profile of the OMVs from the CVOM-2 strain lacked the abundant 100 kDa band (Figure 1B), showing that this band does correspond to the RsaA protein. Comparing the lines corresponding to CVOM-1 and CVOM-2, we observed a higher concentration of the rest of the proteins present in the CVOM-2 sample and the appearance of additional bands (Figure 1B). Since RsaA is the most abundant protein in CVOM-1 vesicles and the same amount of total protein was loaded in both lines, the absence of RsaA in the CVOM-2 vesicles strongly modifies the relative concentration of the other proteins present in the sample, likely resulting in the changes observed in the protein profiles. To better compare the protein profiles of the OMVs produced by these two strains, a fixed protein concentration of the CVOM-1 vesicles was compared to decreasing concentrations of CVOM-2 vesicles (Figure 1C). A comparison of the intensity of the most abundant proteins between the samples revealed an increase of the relative concentration of these proteins of around 3 times in CVOM-2 OMVs. Only slight differences in the intensity of other proteins were detected and only a new protein with a higher apparent molecular weight than RsaA was observed in the CVOM-2 samples (Figure 1C).

### Absence of RsaA Increases Protein Content in OMVs

To compare the OMV yields of the CVOM-1 and CVOM-2 strains, we purified the OMVs from four independent biological replicates and quantified the total protein content by a Bradford assay. We decided to include in this experiment the *ompA2* null mutant since this is a frequently used strategy to increase OMV production. In all cases, the OMVs were purified from the same culture volume and resuspended in the same final volume (Figure 2A). The protein content in the OMV samples from the strain lacking RsaA (CVOM-2) was 2.5 and 5 times that of the CVOM-1 and the CB15N wild-type strains, respectively. The samples from the *ompA2* null mutant had the same protein content as the CVOM-1 strain (Figure 2A); however, staining these cells with FM4–64FX showed an increase of larger OMVs for this strain (Figure S1 of the Supporting Information). Deleting the *rsaA* gene in a wild-type background did not result in a significant increase in the protein yield, indicating that the effect observed in the CVOM-2 strain results from the combination of the *ompA2*_R351A_ allele and the absence of the S-layer. To test whether the Δ*ompA2* allele could replace the *ompA2*_R351A_ point mutation in the CVOM-2 strain, we obtained a Δ*ompA2*/Δ*rsaA* double mutant. The OMVs yield of this strain was similar to that of the wild-type or Δ*rsaA* strains (Figure 2A), and accordingly, when the cells of this strain were stained with FM4–64FX, no OMVs could be detected (Figure S1 of the Supporting Information). which the *ompA2*_R351A_ allele induces the production of the OMVs is different from that of the *ompA2* null mutation and does not rely exclusively on the loss of function of this OM stabilizing protein. Additionally, we compared the OMV yield of CVOM-2 with those of wild-type E. coli and Δ*nlpI* mutant strains. Under our experimental conditions, the E. coli Δ*nlpI* mutant produced around two times more OMVs than the wild type but this increment was not significant (Figure 2A); a similar but significant yield increment has been reported for the Δ*nlpI* mutant strain using different OMV quantification techniques.^42,43^ Notably, the CVOM-2 strain produces about four times more OMVs than the E. coli Δ*nlpI* mutant strain. To compare with a different OMV-inducing method, we purified OMVs from wild-type E. coli cells by heat shock following an optimized protocol.^44^ This protocol resulted in a 5-fold yield increase from the E. coli wild-type strain but was still significantly lower than that of the CVOM-2 strain. These results show that in terms of its OMV yield, the CVOM-2 strain is appropriate for its use as an OMV-producing strain.

**Figure 2:**
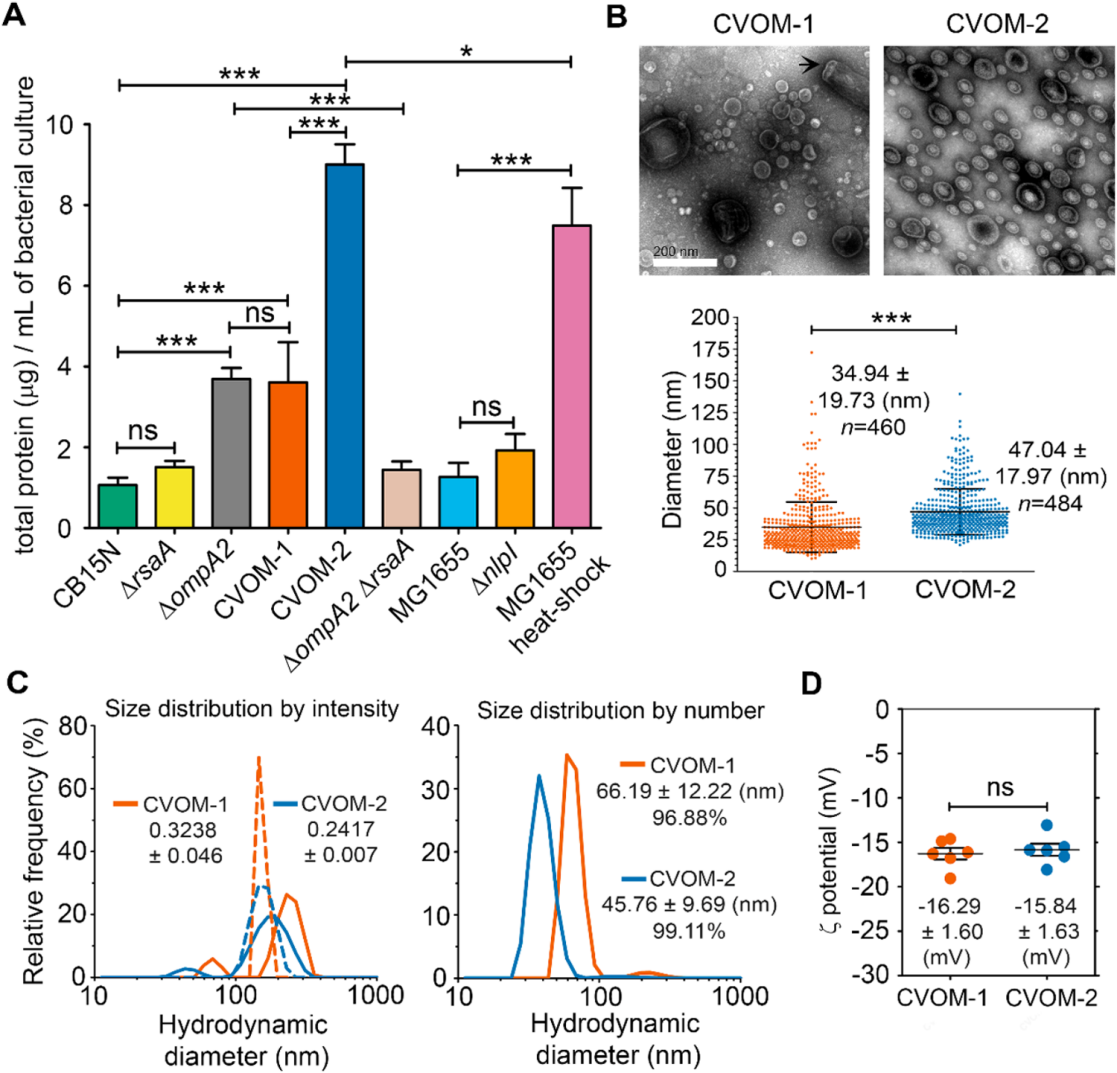
Comparison of OMV yield and OMV characterization. (A) OMV yield of the indicated strains or treatment. OMVs were purified by filtration and ultracentrifugation from four independent biological replicates. Strains: Δ*rsaA* (SP1563), Δ*ompA2* (LDG2), CVOM-1 (SP1565), CVOM-2 (SP-1566), Δ*ompA2*Δ*rsaA* (SP1622), and Δ*nlpI* (SP1567). (B) Scanning electron micrographs showing the morphology and size heterogeneity of OMVs. A broken stalk is indicated by an arrow in the CVOM-1 image. The plot shows the size distribution of the OMVs in the CVOM-1 and CVOM-2 samples. The diameter was measured for 460 and 484 vesicles, respectively. In the case of the CVOM-2, since the vesicles showed an ellipsoidal shape, the area of the ellipse was determined and the diameter of a circle of the same area was calculated. (C) Hydrodynamic diameter (nm) of OMVs from the CVOM-1 and CVOM-2 strains, determined by DLS. Left, the size distribution by intensity is shown and the polydispersity index of both samples is indicated. The discontinuous and continuous lines represent the mean of measurements in which one or two size classes were detected, respectively. Right, the size distribution by number for the mean of two-peak measurements is shown. The mean hydrodynamic diameter ± SD and the relative abundance of the smaller size population are indicated. Two independent samples were mixed first and then analyzed. (D) Plot showing the ζ-potential for CVOM-1 and CVOM-2 vesicles. Each point corresponds to individual measurement repeats for two independent samples. The asterisks indicate the degree of statistical significance between the indicated data sets (*< *p*:*<0.05, **<0.01, ***<0.001).

The size of the OMVs can be relevant for their immunological activity since it affects the uptake pathway by APCs and their dispersion after inoculation.^45–47^ For this reason, we determined the size distribution of the OMVs produced by the CVOM-1 and CVOM-2 strains by transmission electron microscopy (Figure 2B). OMVs isolated from the CVOM-1 strain had a diameter ranging from 10 to 172 nm, with a mean of 34.9 ± 19.8 nm. In contrast, the OMVs from the CVOM-2 strain had a diameter distribution of 21–140 nm with a mean of 47 ± 18 nm. The size distribution indicated that for both strains, about 70% of the OMVs were smaller than 40 nm (Figure 2C). Although the size distribution was similar, we noticed that the OMVs from the CVOM-1 strain showed a tendency to form clumps, and the sample was contaminated with broken stalks (Figure 2B). Determination of the size of the OMVs by dynamic light scattering showed inconsistent distributions between runs for both samples that consisted of two or a single peak (Figure 2C, left graph). This effect was more frequent in the CVOM-1 sample and is likely due to aggregation in the sample resulting in a peak with a higher molecular weight; for this reason, we excluded the measurements with a single peak of high molecular weight. Considering only the reads with two peaks, the CVOM-1 samples had a higher polydispersity index than those of the CVOM-2, 0.32, and 0.24. When intensity percent was converted to size distribution, it can be observed that both samples have two peaks; the first peak consisted of 96.88 and 99.11% of the OMV for the CVOM-1 and CVOM-2 samples, with an average size of 66.19 and 45.76 nm (Figure 2C, right graph). The average size determined by transmission electron microscopy (TEM) and dynamic light scattering (DLS) is very similar for the OMVs obtained from the CVOM-2 strain, but there is a discrepancy of around 30 nm for the OMVs from the CVOM-1 strain. It has been reported that coating liposomes and micelles with protein increases their hydrodynamic radius^48,49^ and in the case of the OMVs from the CVOM-1 strain, the presence of the RsaA protein on the surface of the vesicles could be responsible for the increased size detected by DLS. To better characterize the OMVs from these two strains, we obtained their zeta-potential that is relevant for particle uptake by antigen-presenting cells.^50,51^ Despite the absence of the RsaA protein on the surface of the OMVs produced by the CVOM-2 strain, the ζ-potential was not significantly different between the two samples, both showing a value of around −16 mV (Figure 2D). These results indicate that the OMVs produced by CVOM-2 have a lower tendency to aggregate and have a smaller hydrodynamic diameter than the OMVs from CVOM-1, suggesting that in addition to the absence of the increased relative concentration of the other proteins present in the vesicle, they will more easily disperse after inoculation and that the purification of OMVs from CVOM-2 should be simpler and with better yield, especially if filtration methods are implemented.

### OMVs from C. crescentus CVOM-2 Are More Efficiently Loaded with a Constitutive Recombinant Antigen in Their Lumen

Heterologous antigens can be introduced into the lumen of the OMVs as a soluble protein or incorporated into the membrane as a fusion with an OMP or a lipoprotein. We previously reported that a protein fusion of OmpA2_R2351A_ with mCherry is stable and is present in the OMVs produced by the LDG12 strain,^39^ but the majority of this protein remains in the cell OM and does not preferentially accumulate in the OMVs. When the periplasmic mCherry was expressed in the CVOM-1, the fluorescence of the cells was dimmer than in the wild type, and this effect was even stronger in the CVOM-2 cells (Figure 1A), where the majority of the fluorescence was in the OMVs. This indicates that the soluble periplasmic mCherry concentrates in the lumen of the OMVs produced by CVOM-2 cells. To corroborate this idea, we quantified by flow cytometry the number of fluorescent OMVs and their fluorescence intensity. For this, four independent OMV purifications of each strain were performed in single samples. This revealed that the OMVs from CVOM-2 had a mean fluorescence value about 7-fold higher than that from the CVOM-1 strain (Figure 3A). To verify this result, we compared by Western blot the quantity of mCherry in the OMV samples from these two strains (Figure 3B). Quantification of the signal corresponding to mCherry showed that in agreement with the mean fluorescence intensity, the OMVs of the CVOM-2 strain have a 7-fold higher concentration of this protein. From the flow cytometry data, we determined the total particle number in the OMV samples, showing that there was only a difference of 1.25 more particles in the CVOM-2 sample when compared to that in CVOM-1, 3.82 × 10^11^ and 3.06 × 10^11^ and particles/mL, respectively (Figure 3A). These results suggest that the higher yield obtained from the CVOM-2 strain is in part due to a higher soluble protein content in each vesicle. We hypothesized that an OMP would not be preferentially loaded into the OMVs produced in the CVOM-2 strain and that its concentration would better reflect the number of vesicles. To test these ideas, we compared the LDG12 strain that expresses the fluorescent OMP OmpA2_R351A_–mCherry fusion with a derivative of this strain that carries the Δ*rsaA* allele. These two strains are essentially the CVOM-1 and CVOM-2 strain with the exception that in both the OmpA2_R351A_ protein is expressed as a fluorescent fusion. Fluorescence microscopy showed that the abundance of OmpA2_R351A_–mCherry was not affected by the absence of RsaA and that the signal intensity and number of OMVs were similar between both strains (Figure 3C, compared with Figure 1A). Indicating that in contrast with the soluble periplasmic mCherry, the absence of RsaA does not affect the concentration of this OMP in the OMVs. To indirectly compare the number of OMVs produced by these two strains, we quantified the signal corresponding to the OmpA2_R351A_–mCherry protein by Western blot. A significant 1.7-fold increment in the strain lacking *rsaA* was observed; this increment is similar to that calculated from the FACS results. These results indicate that the absence of RsaA has a minor effect on the number of OMVs (approximately 1.5-fold) but that for an unknown reason it greatly increases the concentration of periplasmic mCherry (7-fold) and probably other soluble proteins in the vesicles.

**Figure 3:**
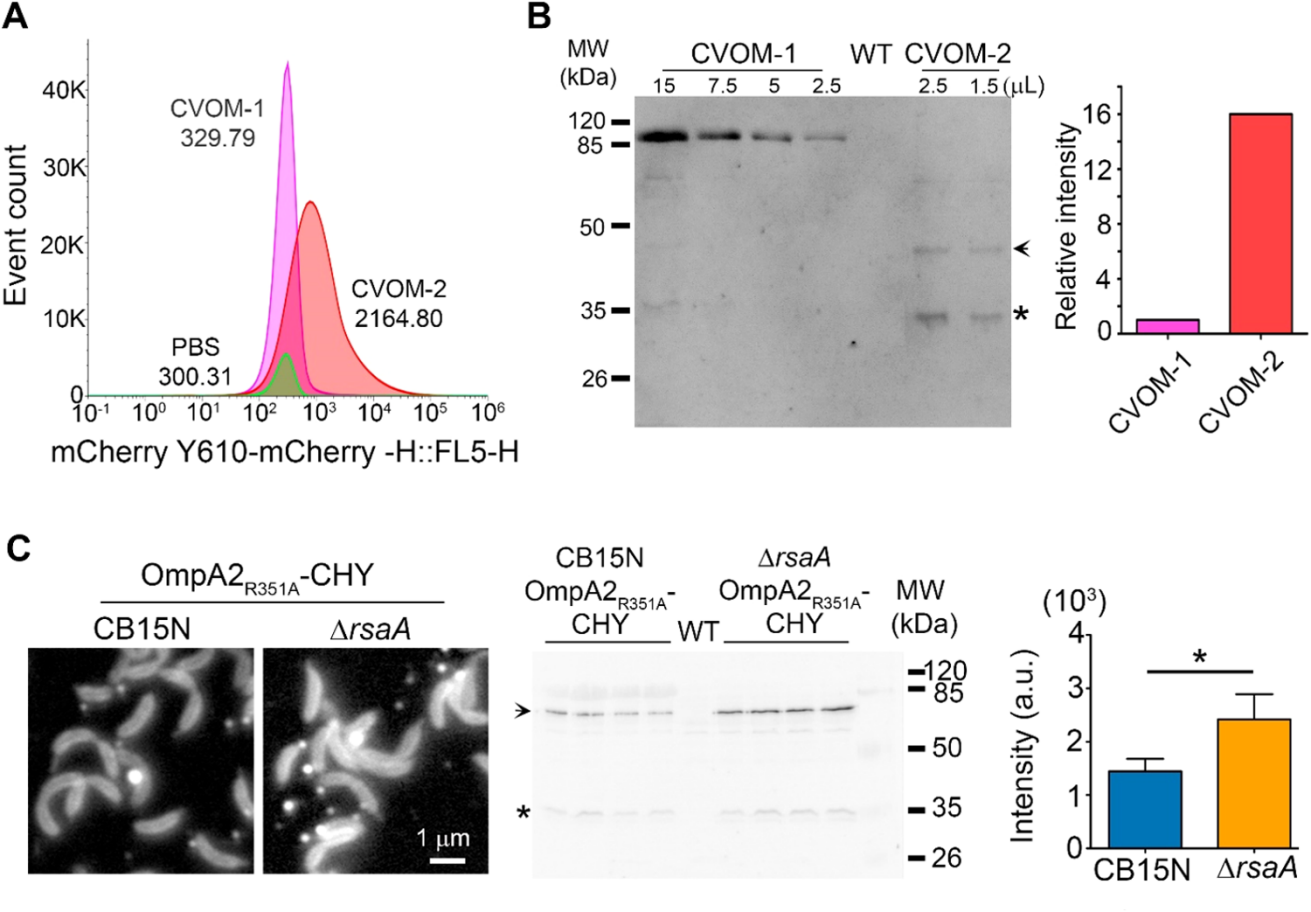
OMVs loading with a recombinant antigen. (A) Flow cytometry plot showing the distribution of the fluorescence intensity of OMVs in four pooled independent purifications from the indicated strains; the mean fluorescence intensity of each sample is indicated. As reference, the PBS vehicle is also included. Strains: CVOM-1 (SP1569) and CVOM-2 (SP1570). (B) Left, comparison of the amount of mCherry present in four pooled independent purifications of OMVs from the indicated strains. Different volumes of the samples were loaded as indicated, and the Western blot was revealed with antibodies against mCherry; right, quantification of the signal corresponding to mCherry in the Western blot. The RsaA protein is visible as an unspecific signal in the CVOM-1 sample; the arrow indicates the mCherry and linker fusion, and the asterisk corresponds to the proteolytic product consisting of only the mCherry (see Figure 1). (C) Comparison of OMV production labeled with a fluorescent OMP. Left: fluorescence micrographs of cells with only the OmpA2 point mutation or with the *rsaA* deletion (similar to CVOM-1 and CVOM-2, respectively). Strains CB15N OmpA2_R351A_-CHY (LDG12) and Δ*rsaA* OmpA2_R351A_-CHY (SP1304); middle: Western blot analysis of the OMVs isolated from LDG12 and SP1304 strains revealed with an antibody against mCherry; four independent purifications were tested for each strain. The arrow indicates the signal corresponding to the protein fusion, and the asterisk indicates the soluble mCherry released by the proteolysis of the fusion protein. Right: quantification of the signal corresponding to the fusion protein expressed in arbitrary units (a.u).

### C. crescentus OMVs Induce Low Acute Inflammatory Response

Due to the presence of LPS and other molecules recognized by the innate immune system, OMVs can show proinflammatory effects. To evaluate the strength of the acute inflammatory response caused by OMVs from the CVOM-2 strain, we measured the induction of the proinflammatory cytokines IL-1β and TNF-α in a widely used murine model of acute inflammation, consisting in the intraperitoneal (i.p.) administration of OMVs and the determination of proinflammatory cytokines in peripheral blood.^52–54^ Mice were administered intraperitoneally with 20 μg of OMVs (about 1 μg per g of body weight) and, for comparison, with 20 μg of OMVs isolated from the E. coli Δ*nlpI* strain. Results are shown in Figure 4, where the amounts of the OMVs are expressed as the protein content in the samples determined by the Bradford assay. As positive and negative controls, we used purified O55:B5 E. coli LPS or PBS solution, respectively. As observed, 2 h post-inoculation (hpi), only LPS and OMVs from E. coli induced the production of IL-1β, whereas mice treated with C. crescentus OMVs showed similar IL-1β levels as the negative control (Figure 4A). For TNF-α determination, blood plasma was collected at 2 and 4 hpi. A significant increase in TNF-α was observed for the E. coli OMVs and LPS samples as early as 2 hpi. In contrast, the OMVs from CVOM-2 only induced a response after 4 hpi, and this was significantly lower than that of the E. coli OMVs at the same time point (Figure 4B).

**Figure 4:**
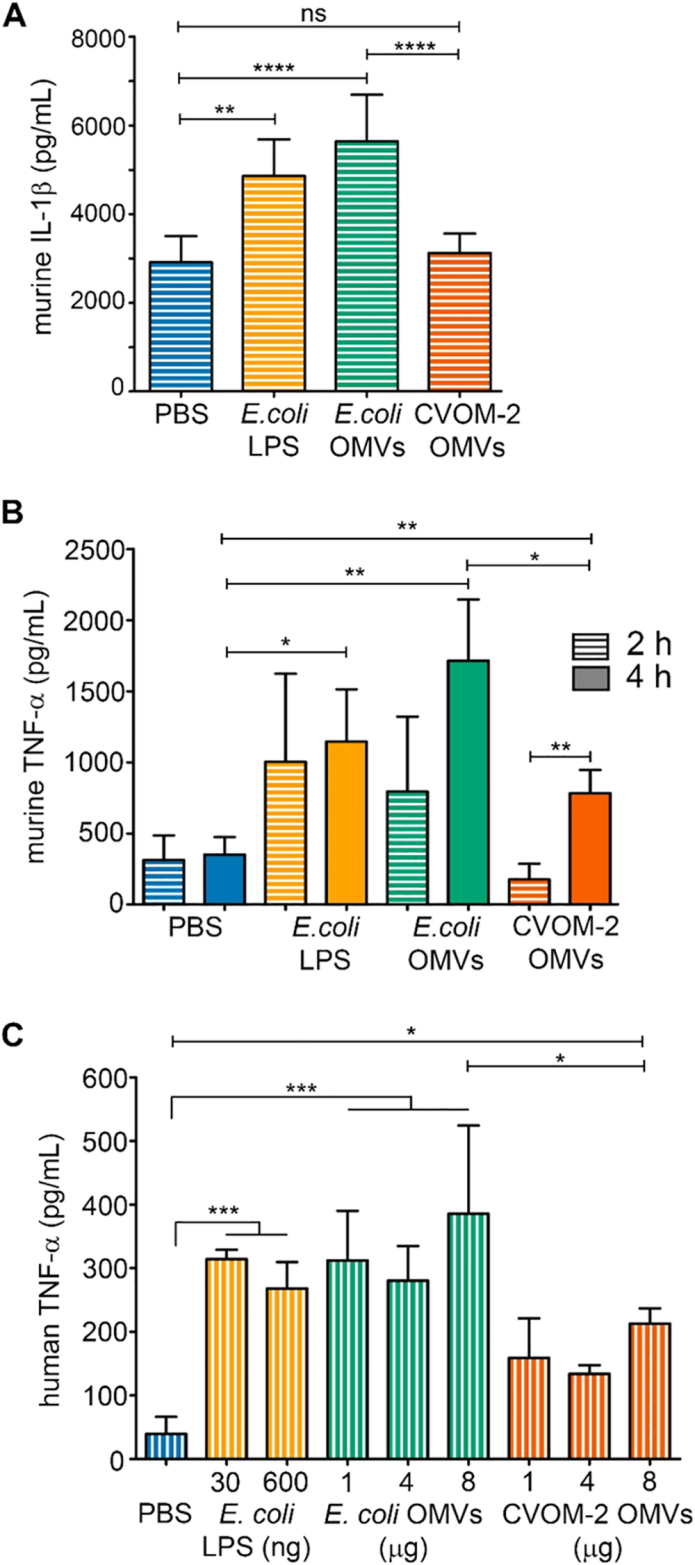
C. crescentus OMVs elicit low levels of the proinflammatory cytokines IL-1β and TNF-α. Panels (A, B), respectively, correspond to the IL-1β and TNF-α levels determined in murine blood plasma at 2 or 4 h post-administration of a single 20 μg dose of OMVs. Statistical analysis were conducted using Welch and Brown–Forsythe ANOVA, followed by Dunnett′s T3 multiple comparison test. Error bars represent SD (*n* = 6 mice). (C) Production of TNF-α by THP-1 human monocytes following exposure to the OMVs for 24 h. TNF-α levels were determined from supernatants of cell cultures, and the data were subjected to statistical analysis using an ordinary one-way ANOVA followed by Tukey′s multiple comparison test. Error bars represent SD (*n* = 3 biological replicates). The asterisks indicate the degree of statistical significance between the indicated data sets (*< *p*: *<0.05, **<0.01, ***<0.001, ****<0.0001).

It has been extensively shown that proinflammatory cytokines such as TNF-α and IL-1β induce neuropathic pain and neuroinflammation leading to alteration in the mouse behavior with well-characterized symptoms.^55,56^ These cytokines are induced not only by the LPS but also by other molecules present in the OMVs such as lipoproteins.^57^ From the third hour after inoculation to the end of the experiment, signs of intense pain were observed in the mice inoculated with the E. coli OMVs or with LPS; these included hunched body, piloerection, reduced mobility, ears back, and exudating closed eyes. In contrast, mice that received C. crescentus OMVs showed normal activity and posture. These observations correlate with the observed lower concentrations of proinflammatory cytokines induced by the CVOM-2 OMVs.

To get a preliminary idea of the safety of the CVOM-2 OMVs in humans, we performed an *in vitro* human monocyte activation test. For this, we exposed human monocytes to different OMVs quantities and measured the production of TNF-α in supernatants. We did not detect a dose-dependent response for any treatment, but similarly to our results with mice, we observed an approximately 2-fold lower response for the CVOM-2 vesicles than that induced by the LPS or OMVs from E. coli (Figure 4C). Taken together, our results show that the OMVs from the *C. crescentus* CVOM-2 strain produce a low inflammatory reaction in mice and human cells.

### OMVs from C. crescentus Induce Antibody Production against a Recombinant Antigen and Have an Adjuvant Effect

The adjuvant effect of OMVs has been widely reported; this property is frequently associated with the activation of the innate immune system and the inflammatory response caused by these vesicles.^58^ Since C. crescentus OMVs induce a low inflammatory response, it was important to assess their capacity to elicit an adaptive response against a heterologous antigen. For this, we immunized BALB/c mice with OMVs from the CVOM-2 strain containing mCherry in their lumen (45 μg of total protein) following previously reported procedures.^25^ To evaluate if these OMVs favored the production of antibodies against the transgenic protein (mCherry) as has been reported for other vesicles, we also inoculated a second group of mice with the purified mCherry protein at a dosage equivalent to that present in the vesicles in the absence of any adjuvant. The concentration of mCherry in the OMVs was estimated by Western blot against a serial dilution of purified mCherry (Figure S2 of the Supporting Information). such as hunching, piloerection, reduced mobility, and exudating closed eyes. Although no inflammatory cytokines were measured during this experiment, the fact that mice inoculated with C. crescentus OMVs only presented slight piloerection, indicates that even at this high dose, the OMVs from CVOM-2 do not induce a strong inflammatory response.

Blood plasma was collected 2 weeks after the final immunization, and the presence of antibodies against the heterologous antigen (mCherry) was tested by Western blot. For this, we used a total cell extract from an E. coli strain expressing OmpA–mCherry from the native promoter of this gene. All of the plasma samples from animals immunized with mCherry-loaded OMVs allowed the detection of the fusion protein; in contrast, plasma from mice inoculated with purified mCherry or saline buffer did not (Figure 5A). A similar experiment using a total cell extract from wild-type E. coli (not expressing the mCherry protein fusion) did not show any difference in protein recognition, only a higher background with the plasma obtained from the mice inoculated with mCherry-loaded OMVs (Figure S3A of the Supporting Information), indicating that the signal observed for OmpA–mCherry was not due to cross reactivity between the proteins present in the CVOM-2 OMVs and in the E. coli cell extract. To show that the signal of the fusion protein was not due to overexpression of this protein, we compared the protein profiles of the strain expressing the OmpA–mCherry protein with that of the wild-type strain. No differences in the region corresponding to the molecular weight of the protein fusion can be observed (Figure 5A, right panel). We also tested the different plasmas against an E. coli cell extract mixed with a known amount of purified mCherry; we only observed a band corresponding to this protein with the plasma from mice inoculated with mCherry-loaded OMVs (Figure S3B of the Supporting Information). These results indicate that C. crescentus OMVs can induce antibody production against a recombinant antigen, even when it represents a minor fraction of the total proteins present in the OMVs. To obtain a quantitative value of the difference in the response against the heterologous antigen when administered within the OMVs or in PBS, we determined the antibody titer using an indirect ELISA assay with the purified mCherry as the antigen (Figure 5B). Only the plasma from mice immunized with OMVs had a titer value above the negative control. For plasma samples from mice immunized with purified mCherry, a consistent but not significant difference from the negative control was observed at low dilutions. These results show that the OMVs from CVOM-2 can induce an adaptive response against a heterologous antigen present in their lumen and that despite the low inflammatory response they have an adjuvant effect.

**Figure 5:**
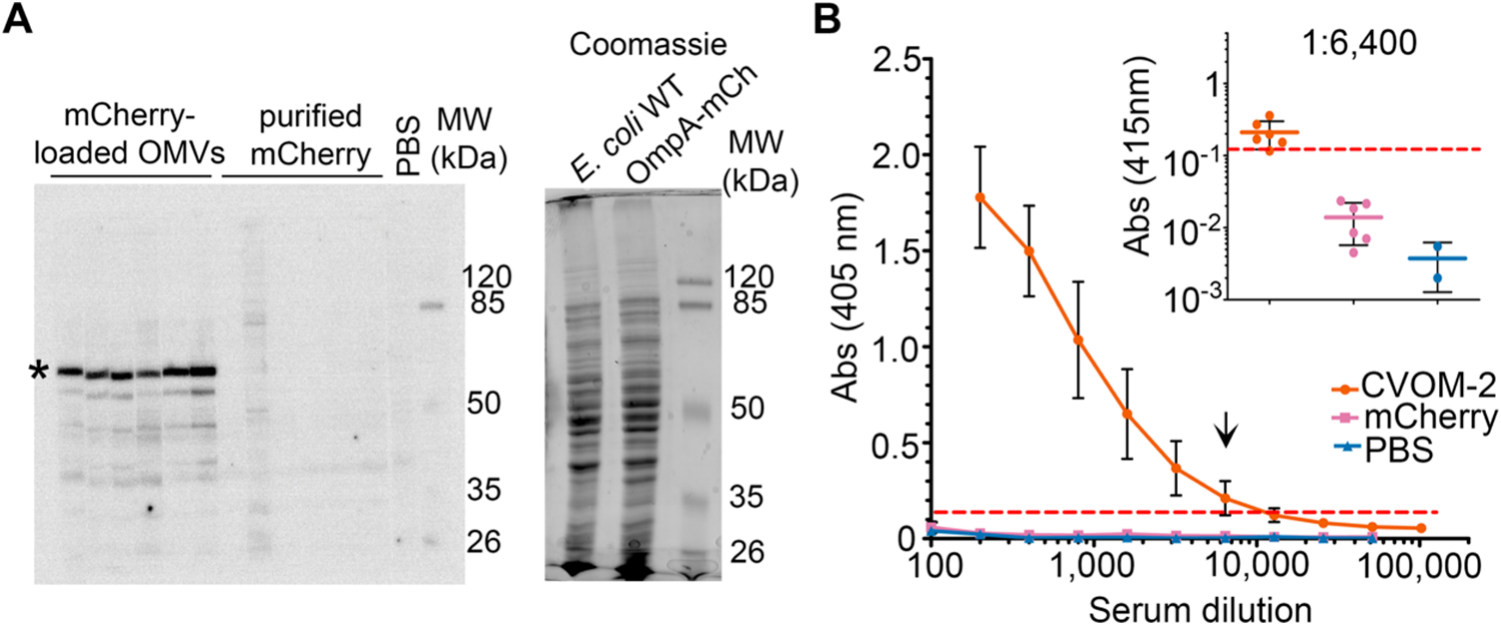
Adaptive response and adjuvant effect against a heterologous antigen contained in CVOM-2 OMVs. (A) Presence of anti-mCherry antibodies in plasma from independent mice inoculated with mCherry-loadedOMVs or with purified mCherry. Left, plasma from individual mice tested in a Western blot against a whole-cell extract from an E. coli strain expressing an mCherry protein fusion (apparent molecular weight ∼ 65 kDa, asterisk); right, Coomassie-stained SDS-PAGE of whole-cell extracts of wild-type E. coli strain MG1655 (E. coli WT) and MG1655 expressing OmpA–mCherry fusion used in the Western blot membrane (OmpA-mCh). Blood plasma from mice inoculated with PBS, purified mCherry, or with mCherry-loaded CVOM-2 vesicles was used at a 1:15,000 dilution, *n* = 6. (B) Antibody titer quantification. The anti-mCherry antibody titer present in the plasma samples used in “A” was determined by an indirect ELISA assay. The plot shows the absorbance at 405 nm from assaying sera at the indicated dilutions. The inset shows data from a 1:6400 dilution, the highest dilution in which positive detection was detected for CVOM-2 sera. The red line indicates the cutoff value calculated according to a statistical method.^59^ Error bars represent SD (*n* = 6).

## Discussion

Due to their natural immunological properties, OMVs have gained significant interest for the development of vaccines. This study introduces C. crescentus as a novel biological system for the production of OMVs with similar yields to those reported for genetically modified E. coli or other bacteria. To increase the production of OMVs, a new strategy was followed that also resulted in a more efficient loading of the vesicles with soluble proteins. Evaluation of the immunological properties of the OMVs produced from C. crescentus showed that they induce a lower amount of inflammatory cytokines in mice and human cells. We found that these OMVs can induce the production of antibodies against a recombinant protein present in the lumen of the vesicles more efficiently than that of the purified recombinant protein. A frequently used target to increase OMV production is the Tol-Pal system; however, these proteins are essential in C. crescentus and many other bacteria. To enhance OMV production in C. crescentus, we used two novel strategies, one consisted of expressing a single amino acid mutant version of OmpA2 that presumably keeps the cell wall binding domain of this protein in a misfolded conformation.^39^ Besides compromising the ability of this protein to interact with the cell wall and to stabilize the OM, this probably also causes periplasmic stress that results in OMV production. The second mutation consisted of deletion of the S-layer protein coding gene. In comparison with a null *ompA*2 mutant, strains carrying the point mutation are more easily obtained^38^ and they produce the same amount of OMVs but with a narrower size range. The deletion of the *rsaA* gene resulted not only in an originally intended increment in the relative concentration of the rest of the proteins present in the OMVs but also in an unexpected more efficient loading of the OMVs with the soluble recombinant protein and probably with other soluble periplasmic proteins. In addition, the number of OMVs produced was also increased by the absence of RsaA. The RsaA protein interacts with the O-antigen of the LPS and forms hexameric complexes that interact with each other, forming a lattice that covers the entire surface of the bacterium.^60,61^ It is not clear why the absence of this structure results in an increase in the periplasmic protein content of the OMVs in the CVOM-2 strain, but it is possible that a physical property of the OM is modified probably resulting in, for example, a change in the speed at which the OMVs grow or their shape before their release. A similar phenomenon has been reported in Salmonella enterica serovar Typhi, where engineered strains carrying different mutations produced OMVs in different amounts and with different lipid-protein ratios.^62^ The RsaA protein has been used for the presentation of epitopes, and our results show that in the OMVs of CVOM-1, RsaA is the most abundant protein. Surprisingly when the deletion of the *rsaA* and *ompA*2 genes was combined, the resulting strain produced less OMVs than the strain carrying the Δ*ompA*2 mutation by itself, showing another advantage of using the *ompA*2 point mutation allele and that the mechanism through which this mutation induces the production of OMVs does not rely on the loss of function of this protein. The comparison of the protein profiles of the OMVs from the CVOM-1 and CVOM-2 strains showed the presence of a high-molecular-weight protein in the CVOM-2 sample (Figure 1C), and this protein was also present in samples from the Δ*rsaA* strain (not shown), suggesting that this protein is induced by the absence or RsaA. From its molecular weight, it is likely that this protein is an integral outer membrane protein that could be stabilizing the OM in the absence of RsaA, similarly to what has been suggested for the BtuB protein in C. crescentus.^63^ Although the removal of the RsaA protein has several advantages, the OMVs from the CVOM-1 strain could serve as an alternative to whole cells for the use of RsaA for the presentation of epitopes.

Characterization of the OMVs produced by the CVOM-2 strain showed that they have an ideal size for vaccine development as antigen carriers since 90% of the vesicles had a diameter of less than 50 nm, making them well-suited for efficient distribution through the lymphatic system and processing by the antigen-presenting immune cells.^12,64^ In comparison with the OMVs produced by different E. coli mutant strains,^25,42^ the OMVs produced by CVOM-2 are approximately 20 nm smaller. This size difference was sufficient to observe differences in the mobilization of liposomes and nanoparticles after injection.^45,65,66^ The CVOM-2 OMVs are in the same size range as those produced by a ΔtolR/galU mutant of Shigella sonnei^25,67^ that have been successfully used for the development of a commercial vaccine. Further work is necessary to determine whether these size differences are immunologically relevant.

The CVOM-2 strain not only efficiently produces OMVs but also can be easily modified to load these vesicles with a recombinant antigen. To achieve this, we developed a system that allows the constitutive expression of heterologous antigens and tested it with a periplasmic version of the mCherry protein. This strategy is cost-effective since it avoids the need for expression inducers, and since the expression vector integrates into the chromosome, the use of antibiotics can be minimized. Although the *rsaA* promoter was used to drive the expression of the transgenic protein, we observed a much lower concentration of mCherry in the OMVs in comparison to the RsaA protein. This could be due to the reported stability of the RsaA coding mRNA,^68^ differences in the codon usage, and stability of the mature protein. Some of these factors can be modified to increase the expression of target proteins.

One of the major challenges associated with the use of OMVs is their biological safety. In comparison to E. coli OMVs, C. crescentus vesicles induced remarkably low inflammatory responses in a murine model and in human cells in vitro. Our results show that CVOM-2 vesicles do not induce the pyrogenic cytokine IL-1β, in accordance with the absence of pain signs in mice. Moreover, the increase in the concentration of TNF-α as a response to CVOM-2 vesicles compared to that to E. coli OMVs was delayed and 2-fold lower. This agrees with the reported low endotoxic potential of C. crescentus lipid A^29^ and suggests that the proteins and other molecules present in the OMVs of the CVOM-2 strain have a low inflammatory potential. Despite their low inflammatory potential, our results showed that the OMVs from the CVOM-2 strain allowed the establishment of an adaptive response against a heterologous antigen, even when it was present in a low relative concentration in the OMVs. The same amount of purified protein did not induce detectable antibodies, indicating a possible adjuvant effect of the OMVs.

The mechanism by which the CVOM-2 OMVs stimulate the adaptative response needs further investigation but it could be caused by a more efficient uptake by immune cells or by the induction of cytokines without triggering a strong inflammatory response as it has been reported for other OMVs.^69,70^ Intraperitoneal administration is a well-characterized model for the study of inflammatory response and has been used to determine the adaptive response to other OMVs and is commonly used for the induction of antibody production.^25,53,71^ However, future studies using other more commonly used vaccine administration routes^72,73^ will allow a better characterization of the immune processes behind the properties of these vesicles.

## Conclusions

The system described in this study not only holds promise as an alternative for vaccine development but also presents a viable option for current therapeutics that rely on C. crescentus whole cells. These encompass both wild-type and engineered strains expressing recombinant proteins, whether in their live or heat-killed states.^31^ While there is evidence supporting that live C. crescentus cells neither replicate nor compromise animal survival when administered to mice,^30,35^ there are reports of C. crescentus species sporadically causing systemic infections in postoperative patients,^74–77^ highlighting the need for caution, particularly in immunocompromised individuals. Although a way to increase the biological safety of therapeutics involving whole C. crescentus cells is killing bacteria by heat inactivation,^31^ OMVs offer the additional advantage that the proteins retain their native conformation, preserving their immunomodulatory potential. Our results support the potential of C. crescentus OMVs as a promising tool for the induction of specific immune responses, opening perspectives for applications in vaccine development.

## Methods

### Plasmids, Bacterial Strains, and Growth Conditions

Strains and plasmids are listed in Table 1 in the Supporting Information, and their construction is described in the text in the Plasmid and Strain Construction section of the Supporting Information. The TOP10 E. coli strain was used to maintain and purify plasmids. These strains were grown in LB medium with the appropriate antibiotic at 37 °C. C. crescentus strains were grown at 30 °C in a peptone-yeast extract (PYE) rich medium. Antibiotics were used at the following concentrations (μg/mL) for E. coli: gentamicin, 20; kanamycin, 50; spectinomycin, 50; nalidixic acid, 20. For C. crescentus, antibiotics were added at the following concentrations (μg/mL) for liquid and solid medium, respectively: gentamicin, 2 and 5; kanamycin, 5 and 20; and spectinomycin, 15 and 100.

### Genetic and Molecular Biology Techniques

All DNA manipulations, analyses, and bacterial transformations were performed according to standard protocols. For a detailed description, see the text and Table 2 in the Supporting Information. Conjugations and transductions were carried out as previously described.^78^

### Fluorescence Microscopy

One microliter of bacterial cultures of the strains expressing mCherry was immobilized on 1.5% agarose pads made with phosphate buffer and imaged by phase-contrast and fluorescence microscopy using an mCherry filter (Chroma Technology Corp, Bellows Falls, VT) in a Nikon Eclipse Ti epifluorescence microscopy. For the staining of the OMV, samples from bacterial cultures at the stationary phase were diluted 1:2 in fresh medium, then stained with the lipophilic dye FM4–64FX (100 μg/mL in DMSO) at a final concentration of 0.5 μg/mL, incubated for 10 min in the dark, and visualized as above.

### OMV Purification

For the determination of the OMV yield, E. coli or C. crescentus strains were cultured in 30 mL of the appropriate media in 125 mL of baffled Erlenmeyer flasks. For mice inoculation experiments, OMVs were purified from 125 mL of cultures grown in 500 mL of baffled flasks. Cultures were grown for 30 h in a water bath at 200 rpm. OMVs were purified by filtration and ultracentrifugation according to previously reported protocols with some modifications.^79,80^ Briefly, cells were harvested by centrifugation at 6500 rpm and 4 °C for 15 min. Supernatants were recovered and vacuum-filtered through hydrophilic-PVDF 0.45 μm filters (Durapore, Merck) and then ultracentrifuged at 35,000 rpm at 4 °C for 2 h. Pellets containing OMVs were washed 3 times with cold phosphate buffer (137 mM NaCl, 2.7 mM KCl, 10 mM Na_2_HPO_4_, 1.8 mM KH_2_PO_4_), pH 7.4, recovered by ultracentrifugation after each washing step, and resuspended by careful vortexing in the same buffer. Finally, for mice inoculation, the aqueous OMV preparations were filtered through hydrophilic-PVDF 0.22 μm filters (Durapore, Merck), and the total protein content was determined by the Bradford assay (Bio-Rad). OMV preparations were stored at −20 °C. For filtration, PVDF membranes were used due to their high flow rates and their lower protein binding in comparison to nylon or nitrocellulose membranes.

### SDS-PAGE and Western Blot Assays

For SDS-PAGE, samples were mixed with sample buffer (final concentrations of 50 mM Tris [pH 6.8], 1% SDS, 10% glycerol, 2% 2-mercaptoethanol, and 2 mM EDTA) and incubated for 10 min at 100 °C. For Western blots, after electrophoresis, proteins were transferred to a 0.45 μm nitrocellulose membrane and incubated overnight with plasma from OMV-immunized mice or with a mouse red fluorescent protein (mRFP) polyclonal anti-RFP antibody raised against 6xHis-tagged mRFP at the dilutions indicated in figure descriptions. For detection, an alkaline phosphatase conjugated to an anti-mouse IgG antibody (1:50,000 vol/vol; Jackson ImmunoResearch) was used together with the CDP-Star/Nitro-block substrate (Thermo Fisher Scientific). Samples were quantified by a Bradford assay (Bio-Rad). Densitometry analysis from Western blots was done by measuring the regions of interest (ROIs) with the integrated density tool in ImageJ.

### Transmission Electron Microscopy

OMVs were isolated as described above from supernatant cultures filtered through 0.45 μm filters. A 5 μL drop of this suspension, containing about 300 ng of OMVs (total protein content), was adsorbed on a Formvar grid for electron microscopy, contrasted by negative staining by using a 4% uranyl acetate solution for 3 min, air-dried for at least 24 h, and visualized with a transmission electron microscope (JEOL 1010, Jeol Peabody, MA) working at 80 kV. Images were obtained with a charge-coupled device CCD camera coupled to the microscope.

### OMV Characterization by Dynamic Light Scattering (DLS)

Purified vesicles were analyzed by DLS, performed with a Zetasizer Ultra (Malvern) equipped with a He–Ne laser (633 nm) as a light source and using a 173° backscatter angle for detection. Data acquisition and analysis were performed with the ZS Xplorer software (Malvern) and applying the viscosity (0.887 mPa·s), the refractive index (1.33), and the dielectric constant (78.5 F/m) values for water as the dispersant and the refractive index of proteins (1.45) as the material type. For size determination, samples at 50–60 μg/mL (protein content) were transferred into a 10 mm path length polystyrene cell (PCS8501) and measured five times at 25 °C with 30 s as the equilibration time. Data processing was carried out with the multiple narrow mode analysis model. The mean hydrodynamic diameter of OMVs was obtained from plots of size distribution by number, which allows its comparison with data obtained by electron microscopy as previously reported.^81^ ζ-potential measurements were performed following the Smoluchowski F­(ka) selection model at 25 °C with 60 s as the equilibration time and using a folded capillary cell (model DTS 1070) filled with 750 μL of sample (30–80 μg/mL of protein content). The measurement process, attenuation, and voltage selection were set as “automatic”, with a minimum and maximum runs of 10 and 100, respectively, and a measurement number of 3 with pauses of 60 s between repeats.

### Flow Cytometry

Purified OMVs from the CVOM-1 and CVOM-2 strains expressing mCherry were diluted 1:100,000 in phosphate buffer, and 100 μL of aliquots was analyzed in a flow cytometer (CytoFLEX LX) with a 610/20 nm bandpass filter for mCherry detection. Data were analyzed using the online software Floreada.io.

### Mice Inoculation

Six week old BALB/c mice (∼20–25 g of body weight) were inoculated intraperitoneally (i.p.) in the lower right abdominal quadrant, verifying the absence of urine, blood, or digesta in the needle hub prior to injection. At the indicated times, mice were terminal bled, and serum or plasma (in the presence of 50 mM EDTA) was recovered by centrifugation. For each treatment and condition, 3 male and 3 female mice were inoculated.

### Murine Model for In Vivo Acute Inflammation

According to reported protocols,^8,82,83^ four groups of mice were inoculated i.p. with 200 μL of PBS, or this solution containing 20 μg of OMVs (derived from E. coli or the CVOM-2 strain) or 20 μg of O55:B5 E. coli LPS (Sigma). Proinflammatory cytokines IL-1-β and TNF-α were determined in plasma after 2 and 4 hpi as markers of systemic inflammation.

### Antibody Production and Determination of OMV Adjuvant Capacity

Six mice were inoculated i.p. twice with 45 μg of CVOM-2 vesicles containing mCherry in the lumen at 2 week intervals and bled 2 weeks after the last dose. For comparison, an independent group was inoculated with purified mCherry at a dosage equivalent to that present in the vesicles. The concentration of mCherry in the OMVs was determined by Western blot using a serial dilution of purified mCherry as a reference.

### Pain Sign Monitoring in Mice

To assess pain, we monitored the facial expression and behavior of treated animals, and these features were scored according to the mouse grimace scale.^84^

### Activation of Human Monocytes by OMVs

THP-1 cells were grown at 37 °C and 5% CO_2_ in RPMI medium, supplemented with 10% inactivated fetal bovine serum (FBS) and 0.05 mM of 2-mercaptoethanol. Cells were seeded in flat-bottom 24-well dishes when cell cultures reached 1 × 10^5^ cells per well; they were challenged with 1–8 μg (total protein content) of OMVs for 24 h at 37 °C. As a negative control, cells were challenged with PBS, and as a positive control with 50 ng mL^–1^ or 1 μg mL^–1^ of O55:B5 E. coli LPS (Sigma).

### Cytokine Determination Assays

Cytokines were measured by ELISA sandwich experiments by using Mini ABTS Development Kits from Peprotech. Briefly, capture antibodies (1 μg/mL) were adsorbed to flat Corning-Costar (3590) polystyrene 96-well plates for ∼16 h at 4 °C in 0.1 M sodium carbonate and bicarbonate buffer, at pH 9.5. The blocking phase was done with a 1% BSA solution in PBS at pH 7.4 for at least 1 h at 37 °C. Standards and samples were added and incubated for 2 h at 37 °C. For cytokine determination in mouse plasma, they were diluted to 1:5 in a 0.3% BSA solution in PBS, while supernatants from monocyte cultures were added without diluting. Plates were washed three times with PBS-0.05% Tween-20 (PBS-T). Detection antibodies (1 μg/mL) were incubated for at least 1 h at 37 °C. Finally, after three additional washes with PBS-T, the cytokine concentration was determined by an enzymatic reaction based on an avidin-HRP conjugate in the presence of ABTS liquid substrate (1 mM in 35 mM citrate-phosphate buffer, pH 4.2). Plates were incubated for at least 30 min at 30 °C for color development, and they were monitored with an ELISA plate reader at 415 nm with a wavelength correction set at 650 nm. When the reaction was near saturation, it was stopped with a 1% (w/v) SDS solution in water.

### Specific Antibody Titration by Indirect ELISA

Purified mCherry (2.5 μg per well) was adsorbed to flat Nunc-Maxisorp polystyrene 96-well plates following the above specifications. Serum samples were serially diluted 2-fold in blocking buffer (containing 0.3% BSA) in a range of 1:100–102,400, added to the wells, and incubated for 2 h at 37 °C. Plates were washed three times with PBS-T, and HRP-conjugated rabbit antimouse IgG antibody (1:5,000; A9044, Sigma) was added to the wells for 1 h at 37 °C. After three additional washes with PBS-T, the ABTS substrate was used for color development. The cutoff value was defined according to a statistical determination method,^59^ considering two negative controls and a confidence level of 95%.

### Ethical Statement

BALB/c mice used in this study were bred and maintained in the animal facilities of the Institute for Biomedical Research, UNAM. All procedures were conducted in accordance with the institutional regulations for the use and care of laboratory animals (protocol ID: 8348).

### Statistics

Data were analyzed with GraphPad Prism, first by a normality test and then by one-way ANOVA followed by multiple comparisons.

## Supplementary Material



## Data Availability

All of the data are available from the corresponding author upon request.

## Notes

OMVs detected by FM4–64FX staining (Figure S1); quantification of mCherry present in OMVs by Western blot (Figure S2); Western blot against E. coli whole-cell extracts revealed with plasma from mice immunized with mCherry in OMVs or in saline buffer (Figure S2); listing strains and plasmids (Table 1); listing the oligonucleotides used (Table 2); and plasmid and strain construction (Text) (PDF)
