# Correction to “Outer Membrane Vesicles from *Caulobacter crescentus*: A Platform for Recombinant Antigen Presentation”

**DOI:** 10.1021/acsnano.5c21718

**Published:** 2026-01-09

**Authors:** Luis David Ginez, Aurora Osorio, Víctor Correal-Medina, Thelma Arenas, Claudia González-Espinosa, Laura Camarena, Sebastian Poggio

This correction is to modify the funding grant number.

