# Supplementary material for: Outer Membrane Vesicles from Caulobacter crescentus: A Platform for Recombinant Antigen Presentation
Source: ACS Nano. 2025 May 28;19(22):20526–38. doi: 10.1021/acsnano.4c17885 (PMC12164514; doi:10.1021/acsnano.4c17885)
Supplement: Supplementary file 1 [file nn4c17885_si_001.pdf]

# Supporting Information

# Outer membrane vesicles from *Caulobacter crescentus*: a platform for recombinant antigen presentation

Luis David Ginez[1], Aurora Osorio[1], Víctor Correal-Medina[1], Thelma Arenas Rodríguez[1], Claudia González-Espinosa[2], Laura Camarena[1] and Sebastian Poggio*[1]

[1] Departamento de Biología Molecular y Biotecnologia, Instituto de Investigaciones Biomédicas, Universidad Nacional Autónoma de México. Instituto de Investigaciones Biomédicas, Circuito Mario de la Cueva s/n, Ciudad Universitaria, Ciudad de México 04510, México.

[2] Pharmacobiology Department and Center for Research in Aging, Center for Research and Advanced Studies (Cinvestav), South Campus.
Calzada de los Tenorios No. 235, Col. Granjas Coapa, Alcaldía Tlalpan, Ciudad de México 14330, México.
*Corresponding author
Sebastian Poggio
Email: sepogh@iibiomedicas.unam.mx
ORCID: 0000-0001-9494-3365

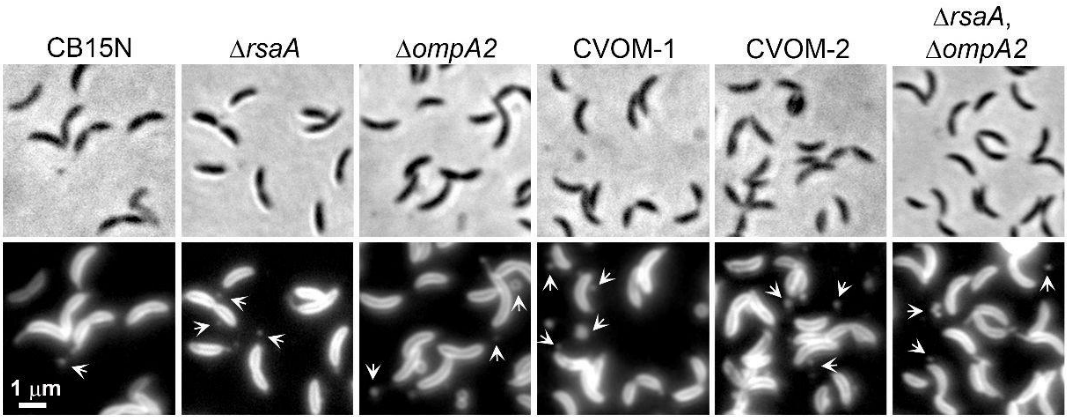


**Figure S1. Determination of presence of OMVs in cultures of different strains by staining with FM4-64FX.** Samples from overnight cultures grown in PYE of the indicated strains were stained with FM4-64FX. Arrows indicate some OMVs, highlighting differences in OMV size diversity, particularly in the ΔompA2 strain.

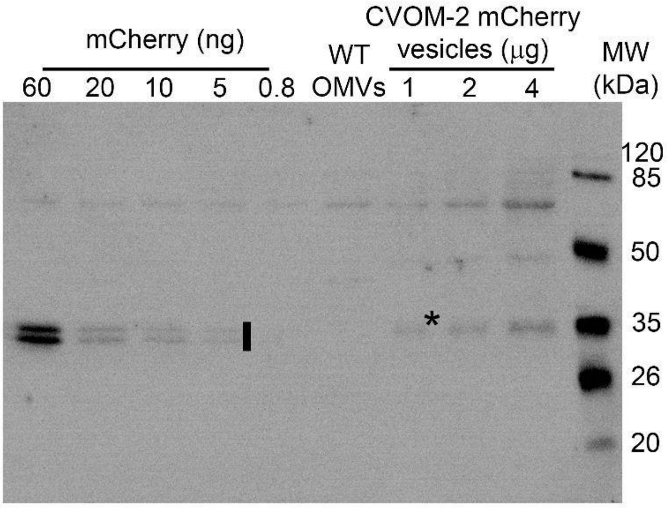


**Figure S2. Quantification of mCherry in CVOM-2 OMVs.** The amount of mCherry in the CVOM-2 OMVs was determined by comparing the signal of different amounts of OMVs against known amounts of purified mCherry in a Western blot revealed with an antibody against mCherry. The indicated amounts of purified mCherry were loaded together with 1μg of purified OMVs not loaded with periplasmic mCherry. The bar and asterisk indicate the signal from the purified mCherry and of the periplasmic mCherry used for quantification.

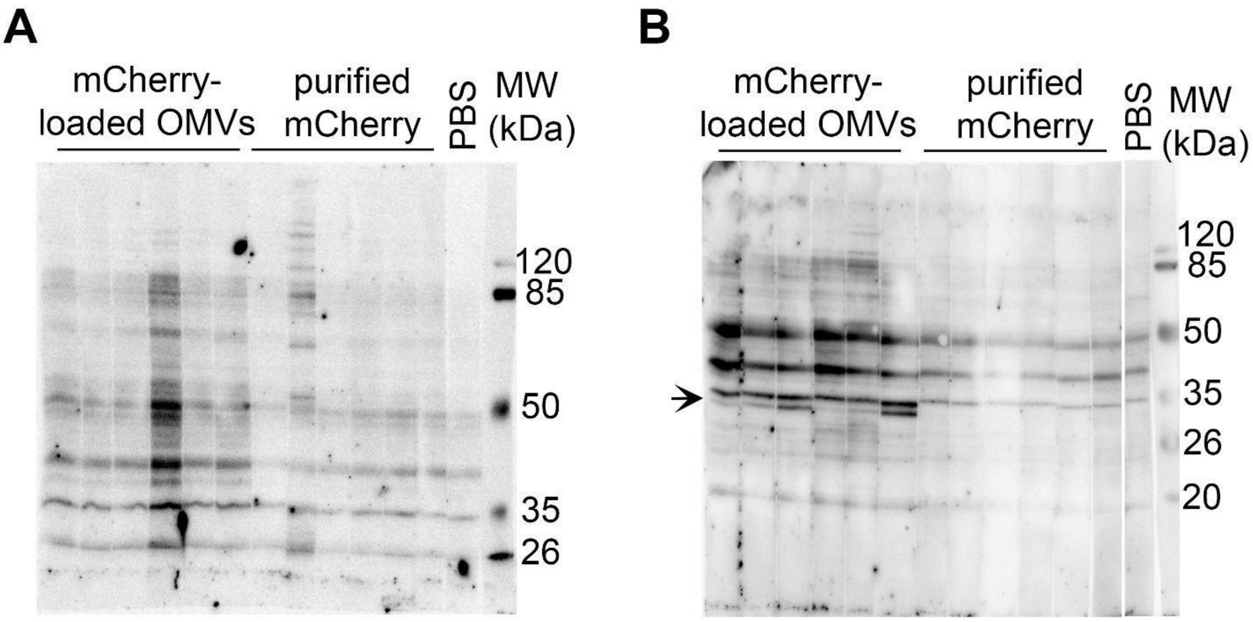


**Figure S3. Adjuvant effect of the OMVs.** The plasma obtained from mice inoculated with the mCherry loaded OMVs, the purified mCherry or with saline solution was tested against E. coli whole cell extracts as negative controls (A) and against purified mCherry mixed with the same whole cell extracts at a concentration of 77 ng per strip (B). To obtain the strips a single well (7 cm) comb was used and then cut in 13 strips, 1μg of purified mCherry was used for the well. The arrow indicates the signal corresponding to the mCherry.

**Table 1. Strains and plasmids used in this work.**

| **Strain or plasmid** | **Description or genotype** | **Reference** |
|---|---|---|
| **Strains** | | |
| CB15N | Synchronizable derivative of CB15 | 1 |
| SP1565 (CVOM-1) | CB15N *ompA2*::pompA2$_{R351A}$-C2 | This study |
| SP1563 | CB15N Δ*rsaA* | This study |
| SP1566 (CVOM-2) | Δ*rsaA ompA2*::pompA2$_{R351A}$-C2 | This study |
| SP1118 | CB15N vanR::rsaAp-SP2-CHY-N4 | This study |
| SP1569 | CVOM-1 vanR::rsaAp-SP2-CHY-N4 | This study |
| SP1570 | CVOM-2 vanR::rsaAp-SP2-CHY-N4 | This study |
| SP1587 | Δ*rsaA* vanR::rsaAp-SP2-CHY-N4 | This study |
| MG1655 | Wild-type E. coli strain, K-12 derivative | |
| SP1567 | MG1655 Δ*nlpI*::ΩKan | This study |
| LDG2 | Δ*ompA2 ompA2*:: ΩSpc | 2 |
| LDG12 | CB15N *ompA2*::pompA2$_{R351A}$-CHYC2 | 3 |
| SP1564 | Δ*rsaA ompA2*::pompA2$_{R351A}$-CHYC2 | This study |
| SP1568 | MG1655 *ompA::*ompA-mCherry | This study |
| SP1622 | Δ*ompA2 ompA2*:: ΩSpc, Δ*rsaA* | This study |
| | | |
| **Plasmids** | | |
| pompA2$_{R351A}$C2 | pompA2 carrying the substitution of R351 codon for A. Resistance for kanamycin. | This study |
| pNPTSΔrsaA | pNPTS138 carrying ΔrsaA. Resistance for kanamycin. | This study |
| pV-rsaAp-SP2-CHY-N4 | pVCHYN-4 derivative carrying the rsaA promoter and the signal peptide of DipM. Resistance for gentamycin. | This study |
| pompA2$_{R351A}$-CHYC2 | pompA2-mCherry carrying the substitution of R351 codon for A. Resistance for kanamycin. | 3 |
| pTRC-*Ec*ompA | pTRC derivative carrying the *E. coli ompA* coding sequence in frame with mCherry and the cat resistance cassette. Resistance for ampicillin and chloramphenicol. | This study |
| pVCHYN4 | pMB1 replicon carrying *vanR, vanA*p, and *mCherry.* Resistance for gentamycin. | 4 |
| pVsp2CHYN4 | pVCHYN-4 derivative carrying the signal peptide of DipM. | 5 |
| pNPTS138 | pLitmus derivative carrying *oriT* and *sacB.* Resistance for kanamycin. | MRK Alley, unpublished |
| pMXFL1 | pSU311 derivative carrying mCherry coding sequence and a kanamycin resistance cassette flanked by FRT sites for FLP-mediated recombination. | 6 |

| | | |
|---|---|---|
| pMXFL2 | pSU311 derivative carrying mCherry coding sequence and a chloramphenicol resistance cassette flanked by FRT sites for FLP-mediated recombination. | 6 |

**Table 2. Oligonucleotides**

| Name | Sequence |
|---|---|
| mCh stop F EcoRI | caa *gaattc* aagtaagctagctgcagc |
| ompA2 stop R EcoRI | caa *gaattc* tta gaagttgatcgagatcgtc |
| delrsaA F3 BamHI | caaa *ggatcc* ggcgttcgagctgct |
| delrsaA R1EcoRI | caa *gaattc* ttgtctcccaaaaaaaatcccacacc |
| delrsaA F2 EcoRI | caa *gaattc* cgccaccgaagtcctgacgctcg |
| delrsaA R4 SpeI | caaa *actagt* ggccgagatcttgccg |
| rsaApr F1 SphI | caaa *gcatgc* cttgcacctccgaggcgctgc |
| rsaApr R1 NcoI | caa *ccatgg* aggattgtctcccaaaaaaaatcccac |
| DipMspF2 BspHI | caaa tcatga ggcagttgtggacgcaagc |
| NfusionR1 | tggccgctctagaactagtggatcc |
| nlpI REC F1 | ggacgttcattcaaccgtggtcttcgggagtgggaaatgaagaagcttgtgtag gctgg |
| nlpI REC R1 | ggctgatgtgtacgtcagctattgctggtccgattctgctgcagatcatcgagctct cc |
| nlpI del F1 | gcagatgggtcaggaagtacc |
| nlpI del R1 | ttcgaattcagccatgtagtacg |
| ompAF2 EcoRI | caa *gaattc* aaaaagacagctatcgcgattgc |
| ompAR3 SacI | caa *gagctc* c agcctgcggctgagttacaac |
| mChFRTF SacI | caa *gagctc* cg atggtgagcaagggcgag |
| mChFRTR EcoRI | gatc *gaattc*atatgaatatcctccttag |
| ompA REC F1 | gatcgaagttaaaggtatc |
| ompA REC R1 | aaaaccccgcagcagcggggtttttctaccagacgagaacgaacttcggcgc gcctac |

### Plasmid and strain construction

pompA2$_{R351A}$ was constructed from the pompA2$_{R351A}$-CHYC2 plasmid by amplifying by PCR using primers mCh stop F EcoRI and ompA2 stop R EcoRI to eliminate the coding sequence for mCherry and incorporating a stop codon at the 3’ of ompA2.

To construct pNPTSΔrsaA, the PCR products amplified from CB15N chromosomal DNA with primer pairs delrsaA F3 BamHI/delrsaA R1 EcoRI and delrsaA F2 EcoRI/delrsaA R4 SpeI were digested with EcoRI, ligated and the ligation product was used as template in a new PCR reaction with primers delrsaA F3 BamHI/delrsaA R4 SpeI. The resulting product was cloned in the EcoRV site of pNPTS138.

To construct pV-rsaAp-SP2-CHY-N4, the PCR products obtained with primers rsaAprF1 SphI/rsaApr1 NcoI and DipMspF2 BspHI/NfusionR1 from CB15N chromosomal DNA or pVsp2CHYN4 as templates were digested with NcoI and BspHI respectively, ligated and the ligation product was used as template in a new PCR with primers rsaAprF1 SphI and NfusionR1. The resulting product was digested with SphI and NheI and cloned with the fragment of pVsp2CHYN4 plasmid digested with the same restriction enzymes.

To construct plasmid pTRC-*Ec*ompA, the PCR products obtained with primers ompAF2 EcoRI/ompAR3 SacI and mChFRTF SacI/mChFRTR EcoRI from E. coli chromosomal DNA or pMXFL2 as templates respectively, were digested with SacI, ligated and the ligation product was used as template for a new PCR with primers ompAF2 EcoRI/mChFRTR EcoRI. The resulting product was digested with EcoRI and cloned into pTRC99a plasmid digested with EcoRI.

SP1563 (Δ*rsaA*) strain was obtained by electroporation of CB15N cells with pNPTSΔrsaA, transformants that integrated the plasmid in the chromosome were selected by plating on PYE plates containing kanamycin. Excision of the plasmid and deletion of the *rsaA* allele was selected by plating on PYE plates containing 3% sucrose followed by replica plating in PYE-Kan and PYE plates. Deletion of the *rsaA* allele was verified by PCR using primers delrsaA F3 BamHI/delrsaA R4 SpeI.

SP1565 (CVOM-1) and SP1566 (CVOM-2) strains were obtained by integrating the pompA2$_{R351A}$C2 plasmid by conjugation into CB15N and ΔrsaA cells. Selection was carried out on PYE plates containing kanamycin.

SP1118, SP1569, SP1570 and SP1587 strains were obtained by integrating the pV-rsaAp-SP2-CHY-N4 plasmid by conjugation into chromosome of CB15N, SP1565, SP1566 and SP1563 strains, respectively. Selection was done on PYE plates containing gentamycin.

To obtain SP1564 strain, plasmid pompA2$_{R351A}$-CHYC2 was incorporated into chromosome of Δ*rsaA* cells (SP1563 strain) by conjugation. Selection was done on PYE plates containing kanamycin.

SP1567 (Δ*nlpI*) strain was obtained by λ-red recombination. MG1655 cells expressing the λ-red proteins were transformed with the kanamycin resistance cassette amplified from pMXFL1 plasmid with primers nlpI REC F1/nlpI REC R1. Selection was done on LB plates containing kanamycin. Allelic replacement was verified by PCR using primers nlpI del F1 and nlpI del R1. Similarly, SP1568 strain was obtained by electroporation of the PCR product amplified with primers ompA REC F1 and ompA REC R1 from plasmid pTRC-*Ec*ompA as template, and selection was done on LB plates containing chlorampenicol.

SP1622 (Δ*ompA2* Δ*rsaA*) was generated by deleting the *rsaA* allele from the LDG2 strain. OMV production was monitored using FM4-64FX staining before and after the gene deletion.

## REFERENCES


(1) Evinger, M.; Agabian, N. Envelope-Associated Nucleoid from *Caulobacter Crescentus* Stalked and Swarmer Cells. *Journal of bacteriology* **1977**, *132* (1), 294–301.

(2) Ginez, L. D.; Osorio, A.; Poggio, S. Localization of the Outer Membrane Protein OmpA2 in *Caulobacter Crescentus* Depends on the Position of the Gene in the Chromosome. *Journal of bacteriology* **2014**, *196* (15), 2889–2900. https://doi.org/10.1128/JB.01516-14.

(3) Ginez, L. D.; Osorio, A.; Camarena, L.; Poggio, S. Establishment of a Protein Concentration Gradient in the Outer Membrane Requires Two Diffusion-Limiting Mechanisms. *J. Bacteriol.* **2019**, *201* (1), 17. https://doi.org/10.1128/JB.00177-19.

(4) Thanbichler, M.; Iniesta, A. A.; Shapiro, L. A Comprehensive Set of Plasmids for Vanillate- and Xylose-Inducible Gene Expression in *Caulobacter Crescentus*. *Nucleic acids research* **2007**, *35* (20), e137. https://doi.org/10.1093/nar/gkm818.

(5) Poggio, S.; Takacs, C. N.; Vollmer, W.; Jacobs-Wagner, C. A Protein Critical for Cell Constriction in the Gram-Negative Bacterium *Caulobacter Crescentus* Localizes at the Division Site through Its Peptidoglycan-Binding LysM Domains. *Molecular Microbiology* **2010**, *77* (1), 74–89. https://doi.org/10.1111/j.1365-2958.2010.07223.x.

(6) Guzmán-Flores, J. E.; Alvarez, A. F.; Poggio, S.; Gavilanes-Ruiz, M.; Georgellis, D. Isolation of Detergent-Resistant Membranes (DRMs) from Escherichia Coli. *Anal. Biochem.* **2017**, *518*, 1–8. https://doi.org/10.1016/j.ab.2016.10.025.
